# Are depressive symptoms linked to a reduced pupillary response to novel positive information?—An eye tracking proof-of-concept study

**DOI:** 10.3389/fpsyg.2024.1253045

**Published:** 2024-02-23

**Authors:** Alexandra M. Spaeth, Stephan Koenig, Jonas Everaert, Julia A. Glombiewski, Tobias Kube

**Affiliations:** ^1^Department of Psychology, University of Kaiserslautern-Landau, Landau, Germany; ^2^Department of Medical and Clinical Psychology, Tilburg University, Tilburg, Netherlands; ^3^Research Group of Quantitative Psychology and Individual Differences, KU Leuven, Leuven, Belgium

**Keywords:** eye tracking, pupillometry, depression, prediction error, belief updating

## Abstract

**Introduction:**

Depressive symptoms have been linked to difficulties in revising established negative beliefs in response to novel positive information. Recent predictive processing accounts have suggested that this bias in belief updating may be related to a blunted processing of positive prediction errors at the neural level. In this proof-of-concept study, pupil dilation in response to unexpected positive emotional information was examined as a psychophysiological marker of an attenuated processing of positive prediction errors associated with depressive symptoms.

**Methods:**

Participants (*N* = 34) completed a modified version of the emotional Bias Against Disconfirmatory Evidence (BADE) task in which scenarios initially suggest negative interpretations that are later either confirmed or disconfirmed by additional information. Pupil dilation in response to the confirmatory and disconfirmatory information was recorded.

**Results:**

Behavioral results showed that depressive symptoms were related to difficulties in revising negative interpretations despite disconfirmatory positive information. The eye tracking results pointed to a reduced pupil response to unexpected positive information among people with elevated depressive symptoms.

**Discussion:**

Altogether, the present study demonstrates that the adapted emotional BADE task can be appropriate for examining psychophysiological aspects such as changes in pupil size along with behavioral responses. Furthermore, the results suggest that depression may be characterized by deviations in both behavioral (i.e., reduced updating of negative beliefs) and psychophysiological (i.e., decreased pupil dilation) responses to unexpected positive information. Future work should focus on a larger sample including clinically depressed patients to further explore these findings.

## 1 Introduction

People with depression hold a pessimistic view of themselves, the world, and their personal future (Beck, 1964). These negative beliefs influence how people process and perceive the world around them, thus maintaining debilitating symptoms of depression. In order to correct such beliefs, people need to be able to update beliefs based on disconfirmatory experiences. However, various lines of research have converged on the finding that people with depressive symptoms have difficulty updating negative beliefs.

Specifically, recent research on dysfunctional expectations in the context of depression has shown that individuals with depressive symptoms, unlike healthy people, uphold negative expectations despite positive information that contradicts their prior expectations (e.g., Kube et al., 2019c). This difficulty seems to apply only to the integration of novel positive information. The integration of novel negative information appears to be unrelated to depressive symptoms, as indicated by a number of experimental studies on expectation change in depression (for an overview, see Kube, 2023). A similar pattern of difficulties in updating beliefs was observed in a related line of research on interpretation biases and cognitive inflexibility, revealing that depression was related to difficulties in revising negative interpretations of social situations based on novel positive information (Everaert et al., 2017, 2018, 2020, 2021). Evidence for cognitive inflexibility in relation to depressive symptoms has also been provided by other research groups (Miranda et al., 2012; Stange et al., 2016, 2017). Further related lines of research on biased belief updating in depression are research on (lack of) the optimism bias (Garrett et al., 2014; Korn et al., 2014) and selective attention (McCabe and Gotlib, 1995; Gotlib et al., 2004; Joormann and Gotlib, 2007).

One compelling framework to understand biased belief updating is the predictive processing framework (Friston et al., 2014). In this framework drawn from theoretical neuroscience, the brain is not viewed as a passive processor of sensory input. Rather, the brain is assumed to constantly generate predictions about the world, which are compared with incoming sensory input. In the case of prediction errors (PEs), i.e., a mismatch of the prior prediction and new information, predictions are adjusted in order to minimize future PEs. In other words, in healthy human learning, PEs are normally used to correct future predictions (Knill and Pouget, 2004; Huang and Rao, 2011; Clark, 2013; Kanai et al., 2015).

Among others (Barrett et al., 2016; Clark et al., 2018), Kube et al. (2020) proposed a predictive processing model of depression. Combining the clinical with a neuroscience perspective, the authors assume that an imbalance between the weight of prior predictions and new information explains why depressed individuals often do not update their negative expectations in response to positive PEs. Specifically, people with depressive symptoms are thought to give too much weight to negative prior predictions, thereby making it difficult to change them in light of disconfirming information. To make this concept more tangible, it is important to acknowledge that depressive disorders are often (though not always) linked to the experience of negative life events, such as trauma, significant loss, or chronic stress (Monroe and Reid, 2009). As expectations are developed based on previous experiences (Rief et al., 2015), predominantly negative generalized expectations are formed. These negative expectations contribute to an intensified negative perception (Barrett et al., 2016), making it challenging to utilize new positive experiences to modify preexisting beliefs, in the sense of a self-fulfilling prophecy.

Support for this hypothesis mainly comes from neuroimaging studies investigating neural correlates of reward processing in depression. Reward processing can be distinguished into three distinct subtypes: (i) reward liking, which refers to the capacity to experience pleasure in response to rewards; (ii) reward wanting, involving the motivation to engage in behaviors conducive to obtaining rewards; and (iii) reward learning, which relates to using reward PEs signaling differences between expected and received reward to adjust behavior. Each of these subtypes has been linked to abnormalities in diverse neural mechanisms and behavioral deficits associated with anhedonia in depression (Rømer Thomsen et al., 2015; Borsini et al., 2020). Especially with regard to reward learning, a number of functional MRI studies found blunted PE signaling in the ventral striatum for unexpected rewards among participants with depressive symptoms compared to healthy controls (Kumar et al., 2008, 2018; Gradin et al., 2011; Robinson et al., 2012; Segarra et al., 2016). Since the striatum, a component of the basal ganglia, plays a crucial role in encoding reward prediction errors (Garrison et al., 2013), this suggests a neural basis for the impairment observed in individuals with depression regarding the integration of unexpected positive experiences. However, these findings are contrasted by studies that did not find differences between people with depressive symptoms and healthy controls in terms of the reward PE related activation in the ventral striatum (Chase et al., 2013; Greenberg et al., 2015; Rothkirch et al., 2017; Rutledge et al., 2017). This heterogeneity in the literature still leaves some uncertainty regarding the physiological factors underlying the difficulties associated with depression in learning from positive experiences.

Aside from neuroimaging, another well-established method to investigate the psychophysiological mechanisms involved in cognitive processes is eye tracking. Within biological psychology, eye tracking is often used as a method to examine cognitive and emotional processes related to psychological disorders (Nuske et al., 2014; Hepsomali et al., 2017; Mckinnon et al., 2020; Navalón et al., 2021). Concerning the processing of unexpected outcomes, pupillometry is a particularly established method. Pupil dilation has been linked to cortical arousal which seems to be mediated by locus coeruleus–noradrenaline activity transferring information on uncertainty and surprise during decision making (e.g., Aston-Jones and Cohen, 2005). Along these lines, pupil dilation has repeatedly been reported as a marker of surprise about unexpected feedback (Preuschoff et al., 2011; Lavín et al., 2013; de Gee et al., 2021). For example, Satterthwaite et al. (2007) found that the pupil dilation was greater after an unexpected loss compared to the dilation following a more likely loss in a gambling task. This is in support of the hypothesis that pupil dilation signals the processing of an observed difference between expected and actual feedback (i.e., a PE).

Along with other eye movement features (Zhang et al., 2022), pupil dilation has recently gained increasing recognition as a biomarker for depression (Schneider et al., 2020; Yang et al., 2023b; Yang X. et al., 2023). A majority of studies found an association between depression and an increased pupil dilation in response to negatively-valenced stimuli, such as, e.g., sad faces (Burkhouse et al., 2015) or negative words (Steidtmann et al., 2010). A comprehensive examination of the relation between depressive symptoms and pupillary responses to stimuli of varying valence can be found in the systematic review by Yang X. et al. (2023). Moreover, in the field of reward research, pupil dilation has also been discussed as a promising marker of depression. Specifically, Schneider et al. (2020) used a reward anticipation task to compare the pupil dilation of depressed individuals and healthy controls when anticipating monetary reward vs. non-monetary reward vs. no reward. The findings indicated a significant negative correlation between depressive symptom severity and the pupil dilation as a measure of arousal during reward anticipation (Schneider et al., 2020). This could be interpreted as a psychophysiological underpinning for the deficits in reward processing, particularly the discussed aspect of reward wanting, associated with anhedonia in depression (Borsini et al., 2020).

However, with respect to the processing of novel information, research on the association between PE-related pupil reactivity and depressive disorders to date remains relatively limited. Still, a recent pilot study by Guath et al. (2023) investigated the pupil reaction during the receipt of feedback in the context of depressive symptoms. The results provided preliminary evidence of a decreased pupil dilation during the processing of negative PEs, i.e., feedback that is worse than expected, in adolescents with elevated depressive symptoms. The authors explain this finding by referring to the pre-existing negative expectations of people suffering from depression, which lead to less surprise when confronted with a negative outcome (Guath et al., 2023).

Given the heterogeneity of the results of the fMRI studies described above (see Section 1.1), we believe that further research into the psychophysiological underpinnings of the processing of PEs associated with depressive symptoms is necessary. In addition, previous research often used reward learning tasks, which mostly required participants to learn the relationship between abstract visual stimuli and a monetary reward (Rothkirch et al., 2017; Kumar et al., 2018). In order to facilitate the application of such experimental findings to clinical work in potential future studies, it might be beneficial to use stimuli that can easily be transferred to people's everyday lives. Therefore, the aim of this proof-of-principle study was to develop an experimental paradigm which allows us to investigate PE processing in a context that people are well familiar with: the interpretation of social (i.e., interpersonal) situations. After all, besides expectations about the own performance and mood regulation ability, expectations about social interactions play a particularly important role in the development and maintenance of depressive disorders (Kube et al., 2019a). Since the pupil dilation has been identified as a promising biomarker of depression (Schneider et al., 2020; Guath et al., 2023), and it also allows a relatively straight forward investigation of how people respond to PEs (Preuschoff et al., 2011; Lavín et al., 2013; de Gee et al., 2021), we decided to use eye tracking to gain further insights into the psychophysiological mechanisms underlying biased belief updating in relation to depressive symptoms. We therefore adapted a well-established task for the use of eye tracking, in which participants are provided with emotional stimuli from social situations: the emotional Bias Against Disconfirmatory Evidence (BADE) task (Everaert et al., 2018, 2020, 2021). In this task, participants are provided with various descriptions of social scenarios, each of which suggests an initial interpretation of the situation, which can be positive or negative. This initially plausible interpretation is later disconfirmed by additional information. By having participants rate the plausibility of different interpretations after each of three pieces of information, it can be examined how they adjust their interpretations in light of additional information. For the sake of the present study, we made some modifications to the emotional BADE task to make it suitable for the use of eye tracking and PE processing. While we incorporated the “disconfirming-the-negative” scenarios, which initially suggest a negative interpretation that is ultimately disconfirmed, we did not include the “disconfirming-the-positive” scenarios used by Everaert et al. (2018, 2020, 2021). Instead, we designed a novel “confirming-the-negative” scenario type comprising scenarios that initially propose a negative interpretation, which is subsequently confirmed. This allowed us to examine how people respond to unexpected positive as opposed to expected negative information, both behaviorally and psychophysiologically.

At the behavioral level, we aimed to examine the association of depressive symptoms with positive PEs. Specifically, we tested the hypothesis that depressive symptoms are related to a reduced revision of established negative interpretations in response to disconfirmatory positive information. At the psychophysiological level, we investigated the relationship between depressive symptoms and eye tracking parameters. Here, we build on evidence from previous eye tracking research suggesting that the pupil dilation signals surprise about unexpected outcomes (Preuschoff et al., 2011; Lavín et al., 2013; de Gee et al., 2021). Further, we drew on research relating depression to the deficient integration of unexpected positive information (Kube et al., 2019c) as well as blunted PE processing (Kumar et al., 2008, 2018; Gradin et al., 2011; Kube et al., 2020). Synthesizing these findings, we hypothesized that elevated depressive symptoms are related to a smaller pupil dilation in response to disconfirmatory positive information.

## 2 Materials and methods

### 2.1 Participants

In this proof-of-principle study, we used a student sample to investigate the relationship between depressive symptoms and the reaction to new (disconfirmatory) information. Since depressive symptoms also occur without a clinical diagnosis and can still be associated with an impaired quality of life (Bretschneider et al., 2017), we deemed it appropriate to test our new paradigm first in a non-clinical sample. Participants were recruited at the university where the study was conducted. As an incentive for participation, participants either received course credit or financial compensation (20 euros). Exclusion criteria were insufficient knowledge of the German language and a physical condition which disables the person affected to sit still in front of the eye tracker for about 30 min. Since this is a proof-of-concept study, we intentionally opted for a small sample size with the aim of investigating the applicability of the emotional BADE task in the context of eye tracking rather in an explorative than a confirmatory way. Thus, thirty-four participants (19 females and 15 males) took part in the study, most of them (91.2%) being university students. No participants were excluded from the analyses. Their age ranged from 19 to 31 years (*M* = 22.85, *SD* = 3.08). All participants' vision was normal or corrected-to-normal. The study was approved by the local ethics committee (reference number: LEK-310). Each participant gave written consent and was treated according to the ethical guidelines of the German Psychological Society.

### 2.2 Apparatus, software, and environmental conditions

Testing took place in a dimmed laboratory room where the lighting conditions were kept constant with one ceiling light being the only light source. An infrared video-based tower-mounted eye tracker (EyeLink 1000 Plus, SR Research, Canada) was used to record monocular eye movements. Although head-mounted or remote eye trackers offer more comfort and freedom of movement for participants (Yang et al., 2023a), we opted for a tower-mounted eye tracker, which gently restricts the participants' head movements via a forehead and chin rest. We based this decision on the particularly high precision and accuracy of the data obtained by these models, resulting from their greater stability compared to head-mounted ones (Niehorster et al., 2020), while at the same time ensuring only moderate restrictions of participants (Holmqvist et al., 2011). Pupil size and gaze position were sampled at a frequency of 1,000 Hz and the capture of the right vs. left eye was counterbalanced across participants. The calibration of the eye tracker was performed using a nine-point calibration scheme so that a maximum error of < 0.5° was achieved. The eye tracker was table mounted in front of a 27” LCD monitor (ASUS PG278QR, ASUSTeK Computer Inc., Taipei, Taiwan; Zhang et al., 2018) running at 100 Hz. The eye-to-screen distance was 76 cm. Stimuli were presented using Presentation software (www.neurobs.com).

### 2.3 Procedure and experimental design

On arrival, participants were given a brief overview of the study procedures and signed the consent form. They received written instructions and performed a practice trial to ensure that the task was understood. The stimuli presented in the practice trial were similar to the ones of the actual experiment but were not identical so as to avoid preexposure. Before the experiment was started, the eye tracker was calibrated as portrayed above. Some participants had already completed an online questionnaire containing socio-demographic as well as psychometric measures at home before the experiment. The others filled in the brief online questionnaire in the laboratory after the experiment. A brief debriefing followed at the end, where participants were asked about their understanding of the task and were given the opportunity to give feedback.

In our adapted emotional BADE task, participants were presented with a total of 24 scenarios, each of which provided participants with three statements. After each of the statements, which successively provided more information on a scenario, participants were presented with four interpretations of the respective scenario. They were asked to rate the plausibility of each interpretation on a visual analog scale with the anchor statements “very unlikely” to “very likely”. For the analyses, the scores of the visual analog scale were transformed to a scale ranging from 0 (“very unlikely”) to 100 (“very likely”). There were three types of interpretations: Absurd interpretations appeared to be implausible after every statement. Lure interpretations seemed to be the most probable interpretation in the beginning of a scenario but lost plausibility after the third statement. As in previous applications of the BADE task, two separate lure interpretations were presented for each scenario. Since the two lure interpretations were randomly assigned to the subtypes lure 1 and lure 2, we did not expect differences in the plausibility ratings of these two interpretation types. This is why the two lure interpretations were not considered separately for statistical analyses, but were combined into one factor level. Finally, true interpretations were initially less probable but ended up being the most plausible after the third information on a scenario. The valence of each interpretation type varied with respect to the type of the scenario described.

Originally, Everaert et al. (2018) developed two different scenario types for the emotional BADE task, one of which was used in the present study as well: the disconfirming-the-negative (DTN) scenario type. The DTN scenarios start with a negative statement (e.g., “The company you are working for needs to lay off many employees. You are called in to see your boss”). The negative interpretation evoked by the first statement was supported by the second statement which, however, still left some ambiguity concerning the ending of the scenario (e.g. “Your boss looks unhappy when you enter his office”). The third information on the scenario then disconfirmed the initial negative interpretation by suggesting a positive ending (e.g. “Your boss shares how upset he is about having to lay off his employees, and states that he wants you to stay because of your collegiality and achievements”). The two lure interpretations provided for this scenario type were negative (e.g. “Your boss wants you to leave the company because you are not as good as the other employees”; “The boss will have to let you go because you are not a great fit with the team.”) whereas the true interpretation was positive (“The boss wants to keep you in the company because you are one of the better employees”). All 12 DTN scenarios from Everaert et al. (2018) were translated into German. In order to facilitate the measurement of PE processing after the last information on each scenario, the wording of the second statements was slightly modified as compared to the original statements by Everaert et al. (2018). This modification ensured that the positive ending of a scenario was not predictable after the second piece of information, which allowed us to examine participants' responses to PEs.

The second scenario type developed by Everaert et al. (2018) were disconfirming-the-positive scenarios. These scenarios were positive in the beginning but ended with a negative statement disconfirming the initial positive interpretation. Based on the findings of previous studies showing that depression is primarily related to difficulties in integrating unexpected positive information, whereas no such abnormalities were found for the processing of new negative information (Kube et al., 2019b), our primary focus was on participants' reactions to the DTN scenarios. Therefore, we did not use the disconfirming-the-positive scenarios. Instead, in order to be able to investigate whether the unexpectedness (vs. expectedness) of the ending of a scenario (positive vs. negative) has an influence on the pupillary response, we created a new scenario type: confirming-the-negative (CTN). These additional 12 scenarios had a negative beginning like the DTN scenarios, but—unlike the DTN scenarios—also had an unsurprising negative ending, such that we could well investigate how people perceive a surprisingly positive ending in the DTN condition as compared to the CTN condition. Having feasibility considerations in mind (e.g., study duration and participants' fatigue), we decided against a fully-balanced experimental design in this first proof-of-principle study, which is why we did not use any other scenario types in addition to DTN and CTN scenarios.

The CTN scenarios had the same structure as the scenarios developed by Everaert et al. (2018). In designing the content of the scenarios, we drew on the themes of the previous version, thus describing ordinary interpersonal situations involving social rejection and failure. A sample scenario reads: “You are sitting in a café with a friend. Although you feel bad about it because you actually want to lose weight, you order yourself a piece of cake.” (Statement 1), “You see your friend looking at you in irritation as you place your order.” (Statement 2), “Your friend tells you that you should really watch your weight.” (Statement 3). The two lure interpretations were of positive valence (e.g. “Your friend is happy to eat cake with you.”; “Your friend thinks it is good that you eat what makes you happy.”) and the true interpretation was of negative valence (e.g. “Your friend thinks that you should lose weight.”). In order to focus more on the valence of the interpretations, we refer to negative (corresponding to the lure interpretations of the DTN scenarios and the true interpretation of the CTN scenarios) and positive (corresponding to the true interpretation of the DTN scenarios and the lure interpretations of the CTN scenarios) interpretations in the results section.

**Figure 1:**
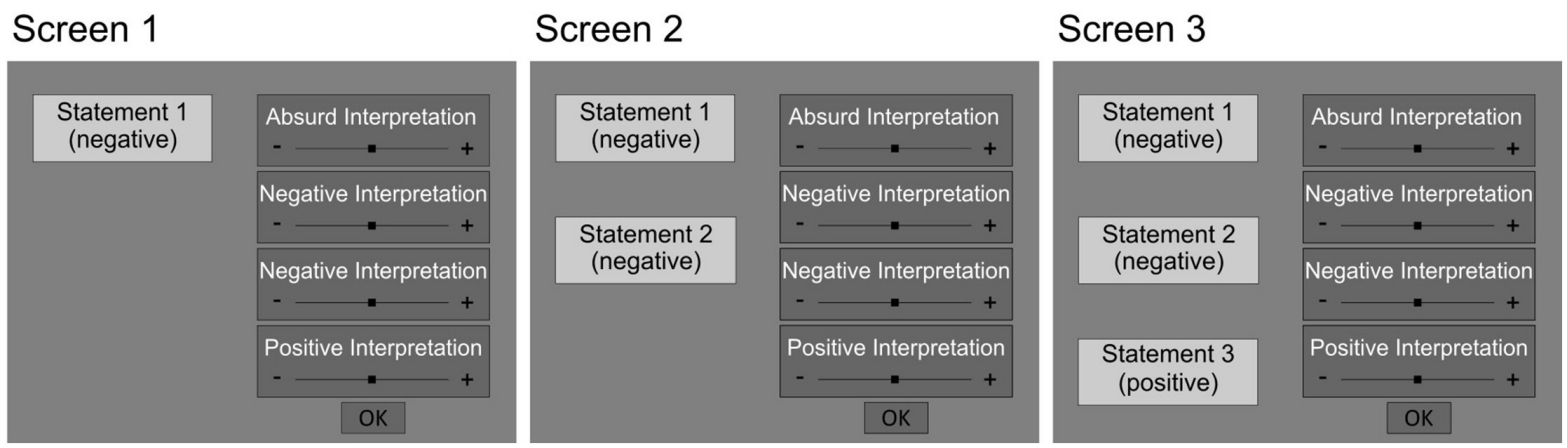
Schematic illustration of the presentation of a DTN scenario. The statements were presented on the left and the interpretations were displayed on the right side of the screen. Participants were instructed to rate the plausibility of each interpretation on the respective visual analog scale after reading the information. By clicking a button, participants could proceed to the next screen presenting the next piece of information on the scenario alongside the familiar interpretations.

All scenarios were self-referential and participants were instructed to imagine themselves in the situation described. Furthermore, the scenarios were presented in a randomized order across participants. The order of the interpretations was randomized across statements and participants. The statements providing information on a scenario were successively displayed on the left side of the screen. The four interpretations of a scenario were continuously presented on the right side of the screen (see Figure 1). The experiment was self-paced, i.e., after reading the information on a scenario and rating the plausibility of the interpretations displayed, participants could click a button to get to the next screen with the next piece of information on the scenario. Concerning the luminance of our stimuli, an essential consideration in eye tracking research, the two experimental conditions (DTN and CTN) did not differ in this respect, as measured at the eye position in our setup. Furthermore, differences in pupil size across experimental conditions were not interpreted *per se*. Instead, we examined how the difference in pupil size across experimental conditions covaries with depression scores. This covariation, in principle, is unaffected by any possible luminance difference between conditions.

### 2.4 Measures

Gender, age, education and employment status were assessed as socio-demographic variables in a brief online questionnaire. The online survey also included the second edition of the Beck Depression Inventory (BDI-II; Beck et al., 1996) in order to measure depressive symptoms. The BDI-II consists of 21 items which asses depressive symptoms experienced within the last 2 weeks on a four-point Likert scale ranging from 0 to 3. In the clinical context, the BDI sum score is used to assess the severity of depressive symptoms. The range of the sum score is between 0 and 63 with higher values indicating increased depressive symptoms. A sum score of ≥ 14 signifies clinically relevant depressive symptoms.

### 2.5 Data processing and statistical analysis

For the analyses of the behavioral data (plausibility ratings), we used a general linear model that included the within-subject factors of Scenario type (DTN, CTN), Information (1, 2, 3) and Interpretation (absurd, negative, positive) as well as the standardized BDI-II sum score as a continuous between-subject covariate. We hypothesized that higher BDI-II sum scores were associated with higher plausibility ratings for the negative interpretations after the third statement in the DTN scenarios.

Regarding the pupil data, we were interested in the pupil dilation elicited by the unexpected positive information at the end of the DTN scenarios. We used a general linear model including Scenario type (DTN, CTN) and Information (2, 3) as within-subject factors. In the DTN scenarios, this captured the change from expected negative information (statement 2) to unexpected positive information (statement 3), while there was no such change in CTN scenarios where both pieces of information were negative. Standardized BDI-II sum scores again were included as a continuous covariate and we tested the hypothesis that participants with higher BDI-II scores exhibited smaller pupil dilation in response to the unexpected positive information at the end of DTN scenarios. All degrees of freedom were corrected using the method of Huynh and Feldt (1976) where appropriate. Type-1 error levels were set at 5%. All statistical analyses were performed using the R software for statistical computing (www.r-project.org).

Before statistical analysis of the pupil data, trials were excluded in which excessive blinking masked more than 10% of the pupil recording (2% of all trials). Remaining eye blinks were replaced by cubic spline interpolation and pupil traces were downsampled to 100 Hz. To capture the changes in pupil size induced by the processing of information 2 and 3, pupil traces were aligned with information onset (when participants performed a mouse click to retrieve the information) and centered at zero (by subtracting the initial pupil size). For each participant, the resulting 48 pupil traces (24 trials with two successive pieces of information each) thus captured changes in recorded pupil size relative to information onset. The recorded traces were then averaged across experimental conditions [Scenario type (DTN, CTN) x Information (2, 3)] to obtain four average traces per participant. The appropriate time window for statistical analysis was given by the reading time of the information (see Section 3.2). We used a velocity-based algorithm implemented in MATLAB (The MathWorks, Inc., 2012) for the preprocessing of eye position traces and the identification and parametrization of ocular fixations (Koenig and Lachnit, 2011; Koenig et al., 2017). More than 99% of the initial fixation clusters on an information had a minimal duration of 900 ms before participants moved their eyes to a different stimulus (the interpretations or previous information). Based on these observed dwell times, statistical analysis was conducted on z-standardized pupil changes from 0 to 900 ms after information onset to prevent any substantial confound of the pupil response with larger eye movements.

## 3 Results

### 3.1 Distribution of depressive symptoms

In our nonclinical sample, BDI-II sum scores ranged from 0 to 34 with a mean of *M* = 7.823 (*SD* = 7.752). The mean sum scores of the BDI-II quartiles were *M* = 0.667 (*SD* = 0.707, *N* = 9) in the first, *M* = 3.857 (*SD* = 0.378, *N* = 7) in the second and *M* = 6.625 (*SD* = 1.768, *N* = 8) in the third quartile. In the last quartile, BDI-II scores ranged from 13 (minimal depressive symptoms) to 34 (severe depressive symptoms) with a mean of *M* = 18 (*SD* = 6.128, *N* = 10). 70% of the sum scores in the last quartile fell within the range of mild depressive symptoms (sum scores between 14 and 19). To explore the influence of depressive symptoms on the probability ratings of the interpretations and on the pupil dilation during the processing of the social information in the BADE task we included z-standardized BDI-II scores as a continuous covariate in our statistical models.

### 3.2 Behavioral data

**Figure 2:**
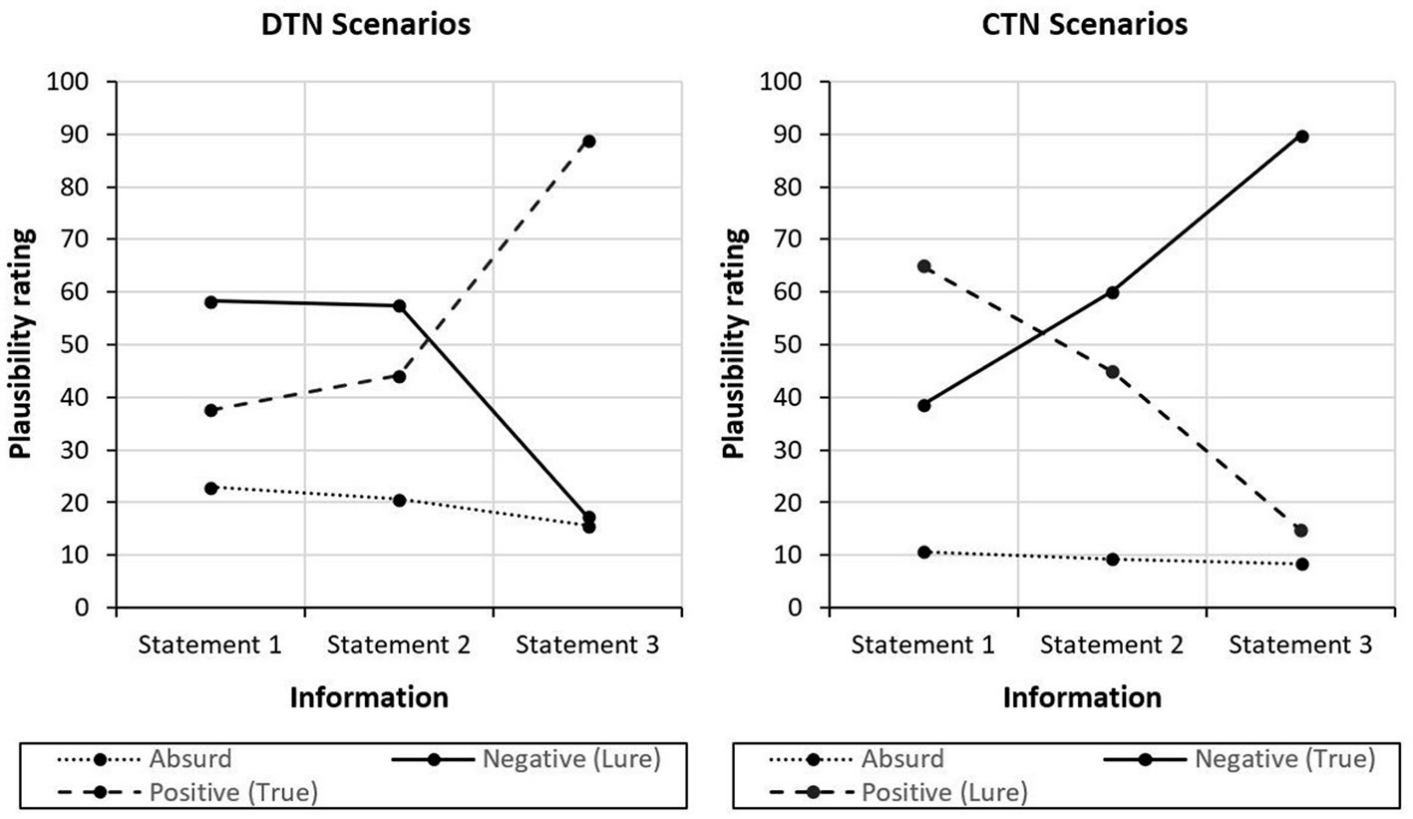
Changes in plausibility ratings of interpretations in response to new information on the scenarios. Plausibility ratings of negative, positive and absurd interpretations after new information are depicted. In the CTN scenarios, all three statements are of negative valence. In the DTN scenarios, the first two pieces of information are negative, the final information is positive.

Figure 2 illustrates the changes in the plausibility ratings of negative, positive and absurd interpretations across the three pieces of information for the two scenario types. A general linear model with the within-subject factors Scenario type (DTN, CTN), Information (1, 2, 3) and Interpretation (negative, positive, absurd) and standardized BDI-II sum scores as a continuous between-subject covariate revealed no significant main effects of the covariate, *F*_(1,32)_ = 0.835; *p* = 0.368; $\eta_{p}^{2}$= 0.025, and the factor Information, *F*_(1.713,54.823)_ = 0.97; *p* = 0.374; $\eta_{p}^{2}$ = 0.029. However, there were significant main effects of the factors Scenario type, *F*_(1,32)_ = 16.542; *p* < 0.001; $\eta_{p}^{2}$ =0.341, and Interpretation, *F*_(1.313,42)_ = 166.296; *p* < 0.001; $\eta_{p}^{2}$ = 0.839. In addition, there were significant two-way interactions Interpretation x Scenario type, *F*_(1.995,63.823)_ = 252.274; *p* < 0.001; $\eta_{p}^{2}$ = 0.887, and Interpretation x Information, *F*_(1.659,53.07)_ = 26.333; *p* < 0.001; $\eta_{p}^{2}$ = 0.451, as well as the three-way interaction Interpretation x Information x Scenario type, *F*_(1.85,59.197)_ = 750.015; *p* < 0.001; $\eta_{p}^{2}$ = 0.959. The interaction Scenario type x Information was not significant, *F*_(1.983,63.449)_ = 1.842; *p* =0.167; $\eta_{p}^{2}$ = 0.054.

The significant three-way interaction indicates that the plausibility of positive and negative interpretations was rated differently in response to new information for the two different scenario types: In the DTN scenarios, negative interpretations were rated to be more plausible than the positive interpretations after the first, *t*_(33)_ = 4.597, *p* < 0.001, *d* = 0.788, and the second piece of information, *t*_(33)_ = 3.389, *p* = 0.002, *d* = 0.581. After the positive third statement, however, the positive interpretations were rated to be more plausible than the negative interpretations, *t*_(33)_ = 32.747, *p* < 0.001, *d* = 5.616, which is in line with the intended disconfirming nature of final piece of information in this scenario type. An almost reversed pattern was found for the CTN scenarios: After reading the first negative information, participants rated positive interpretations to be more plausible compared with the negative interpretations, *t*_(33)_ = 6.805, *p* < 0.001, *d* = 1.167. However, negative interpretations became more plausible than the positive interpretations after information 2, *t*_(33)_ = 2.989, *p* = 0.005, *d* = 0.513. After information 3, this effect became even more pronounced, *t*_(33)_ = 27.567, *p* < 0.001, *d* = 4.728. Thus, although the positive interpretations were unintentionally rated to be most plausible at the beginning, the negative interpretations were rated more and more plausible as the negative content of the scenarios unfolded further, consistent with the design of this scenario type. The ratings of the absurd interpretations were not examined any further as they were not relevant to the research question. As expected, the corresponding plausibility ratings were in a low range throughout both scenario types (see Figure 2).

**Figure 3:**
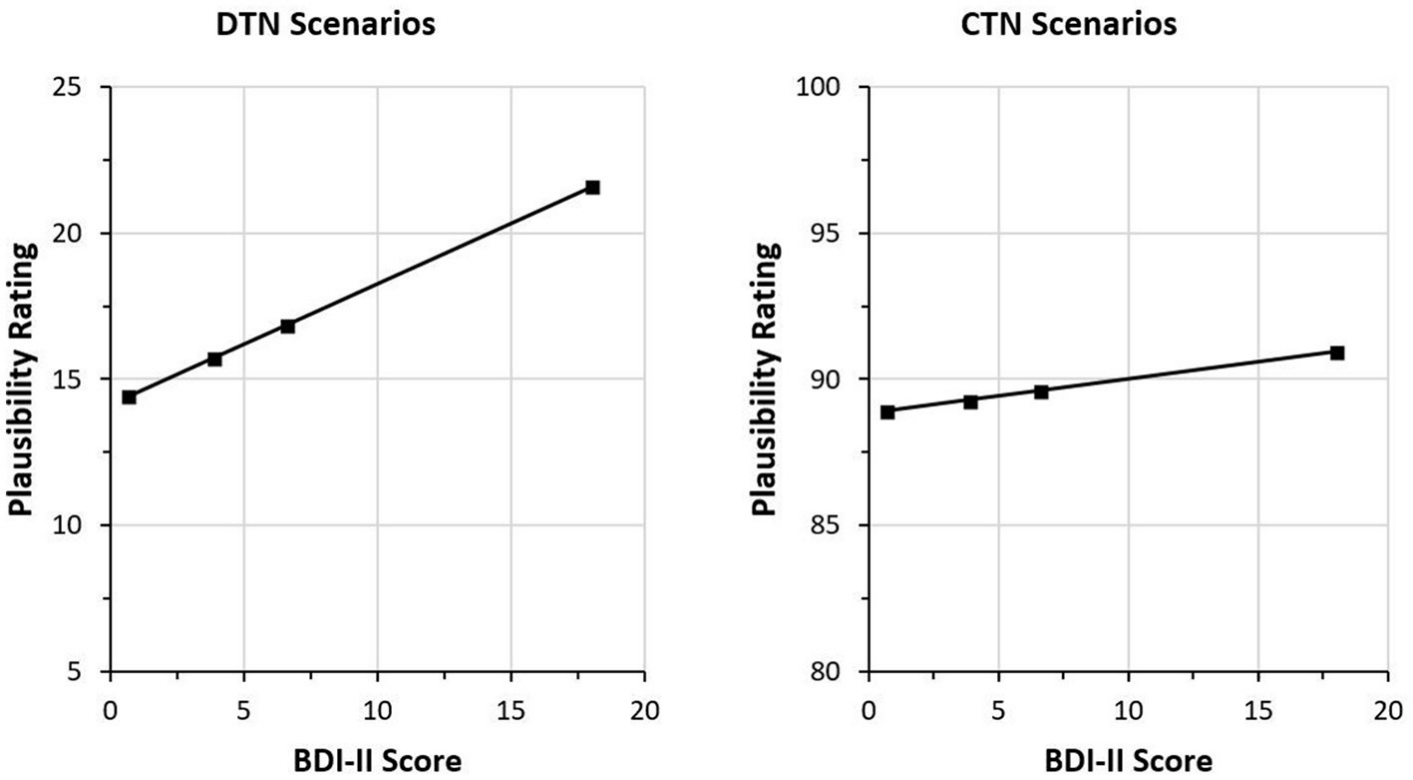
Plausibility ratings of negative interpretations after the third statement as a function of BDI-II sum scores. Higher BDI-II sum scores are associated with higher plausibility ratings of the negative interpretations in both scenario types. In the disconfirming-the-negative scenarios, the third statement is positive and disconfirms the negative impression induced by the initial information. In the CTN scenarios, the third piece of information confirms the negative impression induced by the previous statements. Therefore, the plausibility ratings of the negative interpretations are overall lower in the DTN scenarios. For illustrative purposes, the ratings are shown for the quartile means of the BDI-II sum scores.

Regarding depressive symptoms, no interaction with the other factors was significant (BDI-II x Scenario type, *F*_(1,32)_ = 0.075; *p* = 0.786; $\eta_{p}^{2}$ = 0.002, BDI-II x Interpretation, *F*_(1.312,42)_ = 2.293; *p* = 0.131; $\eta_{p}^{2}$ = 0.067, BDI-II x Scenario type x Information, *F*_(1.983,63.449)_ = 0.013; *p* = 0.987; $\eta_{p}^{2}$ < 0.001, BDI-II x Scenario type x Interpretation, *F*_(1.995,63.823)_ =0.921; *p* = 0.403; $\eta_{p}^{2}$ = 0.028, BDI-II x Information x Interpretation, *F*_(1.659,53.07)_ = 0.621; *p* = 0.512; $\eta_{p}^{2}$ = 0.019, BDI-II x Scenario type x Information x Interpretation, *F*_(1.850,59.197)_ = 0.419; *p* = 0.644; $\eta_{p}^{2}$ = 0.013). Yet, the interaction between depressive symptoms and the factor Information, *F*_(1.713,54.823)_ = 2.771; *p* = 0.079; $\eta_{p}^{2}$ = 0.08, indicated a non-significant trend, which is why we decided to conduct further simple effects analyses to explore the association between the plausibility ratings and depressive symptoms. We considered this an appropriate procedure since the probability for type-II errors increases for small sample sizes (as in the present study). Thus, the chance to find significant effects is substantially reduced in small samples, even if they are moderately sized and potentially meaningful. Indeed, a sensitivity power analysis using G^*^Power (α = 0.05, 1 – β = 0.80, *n* = 34) revealed that only very large effects, *f* = 0.639, could have been detected given the present sample size. Accordingly, we fitted separate general linear models for the three statements comprising the factors Scenario type and Interpretation and the BDI-II covariate. After information 1 and 2, there was no significant effect regarding the BDI-II sum score. After the third information, however, a significant trend indicating an interaction between depressive symptoms and the factor Interpretation, *F*_(1.999,63.97)_ = 3.240; *p* = 0.046; $\eta_{p}^{2}$ = 0.092, was found. This suggests that the three interpretation types were rated differently depending on the BDI-II sum score. Separate analyses for the three interpretation types encompassing the factor Scenario type and the BDI-II covariate revealed a significant trend implying that only the ratings of the negative interpretations were affected by depression scores, *F*_(1,32)_ = 4.216; *p* = 0.048; $\eta_{p}^{2}$ = 0.116. Figure 3 indicates that in both scenario types, the plausibility ratings of negative interpretations increased with higher BDI-II sum scores. The interaction between depressive symptoms and scenario type was not significant, *F*_(1,32)_ = 1.487; *p* = 0.232; $\eta_{p}^{2}$ = 0.044. Correlational analyses showed that, in the DTN scenarios, there was a moderate positive relationship between the BDI-II sum score and the plausibility ratings of the negative interpretations, *r* = 0.384, *p* = 0.025, while no such relationship was found for the CTN scenarios, *r* = 0.114, *p* = 0.52.

### 3.3 Eye tracking data

**Figure 4:**
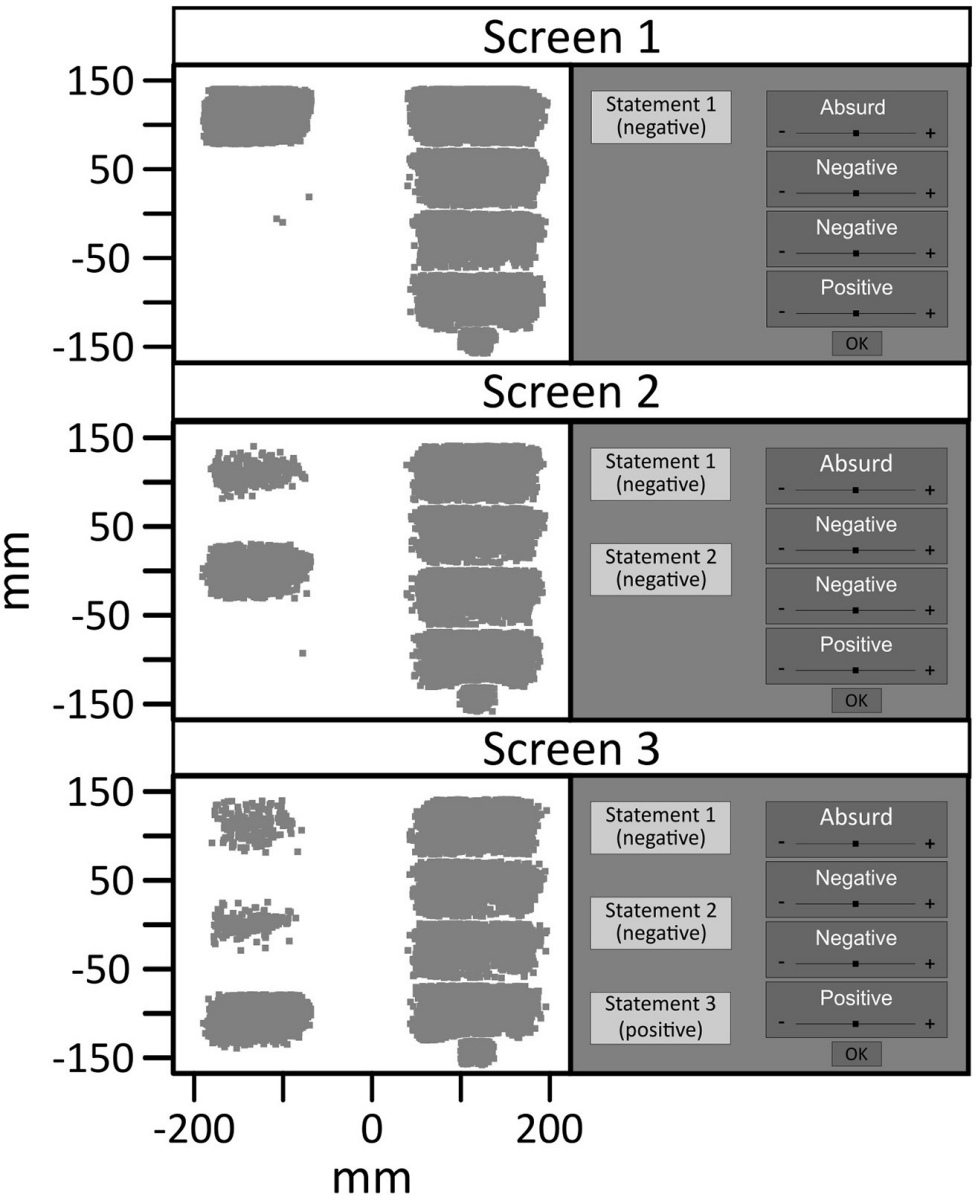
Scatter of fixation positions on the screen with schematic illustration of one scenario. Three pieces of information were successively presented on the left side of the screen. After each new information, the four same interpretations were presented on the right side of the screen. The smaller bottom-right fixation cluster in each panel corresponds to fixations on the OK button that concluded the ratings after each piece of information. Fixations with a distance of more than 6mm to the corresponding square were excluded.

In order to determine the time windows of processing the relevant stimuli, i.e. the three pieces of information on the scenarios, we first examined fixation clusters on the respective stimuli. Figure 4 shows a scatterplot of all fixations on possible target stimuli in the experiment. When reading the description of the scenarios (information 1, 2 and 3), participants moved their eyes to the left side of the computer screen. After each piece of information, participants moved their eyes to the right side of the computer screen in order to read and rate the possible interpretations of the respective scenario.

The start of the information-related pupil dilation was defined by the onset of the information. As participants had to perform a mouse click on the respective field for the information to appear the eyes were on the information initially. The subsequent cluster of fixations on the information had an average duration of 6,245 ms (*SE* = 309.79) for the first, 3,013 ms (*SE* = 172.81) for the second and 2,861 ms (*SE* = 151.88) for third information, respectively. To analyze the pupil response elicited by processing the new information, we were interested in the latest possible time window after the information appeared but before participants disengaged from the information to move their eyes to the interpretations presented on the right side of the screen. More than 99% of the observed fixation clusters on the second and third information had a minimal duration of at least 900 ms before participants moved their eyes to the interpretations. Based on these observed dwell times, statistical analysis was conducted on changes in pupil size from 0 to 900 ms after information onset to prevent any substantial confound of the pupil response with larger eye movements.

**Figure 5:**
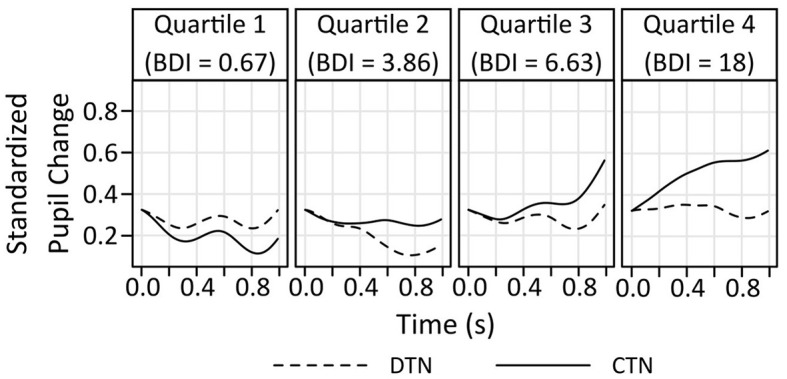
Pupil change in response to the final information on the scenario types as a function of BDI-II sum scores. In the CTN scenarios, this third information is negative, in the DTN scenarios it is surprisingly positive. For illustrative purposes, the pupil change is depicted for the quartiles of the BDI-II. The difference in the pupillary response to the last information of the two scenario types increases with elevated depressive symptoms. Higher depressive symptoms are associated with a smaller pupil dilation to disconfirmatory positive as compared to confirmatory negative information. Of note, comparisons of the absolute pupil size across the different quartiles must not be interpreted since between-subject comparisons are not permissible due to individual baseline differences in the pupil size.

A general linear model that included the factors Scenario type (DTN, CTN) and Information (2, 3) and standardized BDI-II sum scores as a continuous covariate revealed no significant main effect of the covariate, *F*_(1,32)_ = 0.393; *p* =,0.535; $\eta_{p}^{2}$ = 0.012. However, main effects of the factors Scenario type, *F*_(1,32)_ = 4.6361; *p* =,0.039; $\eta_{p}^{2}$ = 0.127, and Information, *F*_(1,32)_ = 22.465; *p* < 0.001; $\eta_{p}^{2}$ = 0.412 were found. In addition, there was a significant trend indicating an interaction between the BDI-II sum scores and the factors Scenario type and Information, *F*_(1,32)_ = 4.239; *p* = 0.048; $\eta_{p}^{2}$ =0.117. Other interactions were insignificant (BDI-II x Scenario type, *F*_(1,32)_ = 3.059; *p* = 0.090; $\eta_{p}^{2}$ = 0.087, BDI-II x Information, *F*_(1,32)_ = 1.075; *p* = 0.308; $\eta_{p}^{2}$ = 0.033, Scenario type x Information, *F*_(1,32)_ = 2.366; *p* = 0.134; $\eta_{p}^{2}$ = 0.069). Analysis of simple effects exploring the impact of the factor Scenario type and the BDI-II sum score within each level of the factor Information revealed that there was no difference in the pupil response to the second (always negative) information in both scenario types, all *F* < 1. In contrast, the pupil did differ in response to the third information as indicated by a main effect of the factor Scenario type, *F*_(1,32)_ = 6.25; *p* = 0.018; η^2^ = 0.163, and an interaction between Scenario type and depressive symptoms, *F*_(1,32)_ = 6.503; *p* = 0.016; η^2^ = 0.169. Figure 5 indicates that the difference in the pupil dilation as a reaction to the final information of the two scenario types increases with higher BDI-II sum scores. The largest difference is visible in the fourth BDI-II quartile, where the pupillary response to the disconfirmatory positive information of the DTN scenarios is smaller than to the confirmatory negative information of the CTN scenarios.

## 4 Discussion

While previous research linked depressive symptoms to difficulties in using novel positive information to revise established negative beliefs, the present proof-of-concept study aimed to examine a reduced pupillary response as a potential psychophysiological mechanism underlying these difficulties. The behavioral data confirm that our adaptation of the well-established emotional BADE task was mostly successful. Replicating previous research (e.g., Liknaitzky et al., 2017; Everaert et al., 2018, 2020), our behavioral data shows that in the DTN condition, depressive symptom severity was associated with a tendency to hold on to negative interpretations of a scenario despite disconfirmatory positive information. Further, in line with previous work (Everaert et al., 2018; Kube et al., 2019b), we found no association between depressive symptoms and the revision of interpretations in response to negative information in the CTN scenarios. The most novel findings are the results of the pupillary response to a surprisingly positive end of interpersonal scenarios, which are discussed in-depth below.

We found that the pupil response to disconfirmatory positive as compared to confirmatory negative information was indeed affected by depressive symptom severity. The difference in the pupillary reaction to the last piece of information on the two scenario types increased with elevated depressive symptoms. Specifically, participants with more pronounced depressive symptoms exhibited a smaller pupil dilation in response to the surprisingly positive information at the end of the DTN scenarios than to the negative information at the end of the CTN scenarios. Since previous eye tracking studies linked the pupil dilation to the processing of unexpected outcomes (Preuschoff et al., 2011; Lavín et al., 2013; de Gee et al., 2021), this finding can be interpreted as a further indication of the recently theorized blunted processing of positive prediction errors (PEs) in depression (Barrett et al., 2016; Kube et al., 2020). It is also in line with the results from a number of fMRI studies pointing to depression being associated with blunted signaling of reward PEs at the neural level (Kumar et al., 2008, 2018; Gradin et al., 2011). Since the amount of surprise, indicated by the magnitude of pupil dilation, has been linked to the amount of learning from expectancy-violating outcomes (Brod et al., 2018), it can be assumed, that individuals with heightened depressive symptoms have a limited ability to learn from surprisingly positive experiences. In terms of the predictive processing framework, our findings suggest that individuals with elevated depressive symptoms afford reduced precision, i.e., weight, to positive PEs (Feldman and Friston, 2010; Kanai et al., 2015). At the same time, it has been proposed that depressed individuals afford too much precision to negative prior beliefs (Kube et al., 2020), which hampers belief updating in light of positive experiences, hence sustaining a depressive mindset. Our results are consistent with that framework as they point to a stronger psychophysiological response to expected negative as compared to unexpected positive information.

An alternative explanation of our eye tracking results could be that the decreased pupil dilation exhibited by people with higher depressive symptoms during the processing of disconfirmatory positive information signals a shallower encoding of this information. As pupil dilation has been linked to a deeper processing and subsequently more successful recognition of verbal stimuli (Papesh et al., 2012; Ariel and Castel, 2014), shallower processing of new positive information could also account for the difficulties of people with depression to integrate positive information that disconfirms their negative beliefs. However, the literature on the association between depth of encoding and pupil dilation is somewhat heterogeneous. Furthermore, pupil dilation has recently been found to reflect time pressure rather than encoding strength (Gross and Dobbins, 2021), which questions this alternative interpretation of our pupillary data.

### 4.1 Limitations

A major limitation of this proof-of-concept study is that the design did not enable us to disentangle the effects of valence and (un-)expectedness of information on the pupillary response. Since the third statement in the DTN scenarios was both positive and surprising, whereas the third information on the CTN scenarios was negative and unsurprising, a comparison of the pupil response to the last information in these two conditions inevitably involves a confounding of valence and (un-) expectedness. Relatedly, the lack of a disconfirming- and a confirming-the-positive control condition also leaves room to alternative interpretations of the difference in the pupillary response to expectation-disconfirming positive vs. expectation-confirming negative information we found in association with higher depression scores. The pupil changes depicted in Figure 5 (see Section 3.3) may also suggest that the increased difference in the pupil response to the final information of the two scenario types shown for participants with higher BDI-II scores may stem from an increased pupil dilation to confirmatory negative rather than a smaller response to disconfirmatory positive information. In a comprehensive review article, Mathôt (2018) divides the psychosensory pupillary response to potentially arousing stimuli into two types: In the first 1,000 ms after presentation of a stimulus, an orienting response emerges, which is particularly strong for stimuli that are unexpected and salient. This orienting response is then followed by slower responses that are associated with arousal or mental effort in processing the stimuli. As an example, Bradley et al. (2008) found that the pupil diameter increases as a function of emotional arousal elicited by emotionally engaging pictures. Following this line, it would be conceivable that our pupillary data indicate an increased emotional arousal of individuals with higher as compared to individuals with lower BDI-II scores when confronted with the negative outcome of a social scenario. Relatedly, other research has shown that individuals with depression, in contrast to their healthy counterparts, showed elevated physiological arousal, manifested through increased skin temperature or altered breathing patterns, in response to unpleasant stimuli, which correlated with an intensified perception of negative emotion (Wenzler et al., 2017). The interpretation of a more pronounced arousal-related pupil response to negative stimuli in depressive populations is further supported by a recent and pivotal meta-analysis by Yang X. et al. (2023). This systematic review summarizes current evidence on pupillary reactivity during the affective processing of negative, positive and neutral stimuli in individuals with depressive symptoms compared to healthy controls. In summary, existing research from this field indicates that individuals with diagnosed depression or higher risk of depression displayed a slightly stronger pupil response to stimuli of negative valence, whereas no group differences were found for positive or neutral stimuli (Yang X. et al., 2023).

Yet, in our study, comparisons of the absolute pupil size across the different BDI-II quartiles must not be interpreted due to potential baseline differences in the pupil size of individuals assigned to the each of the quartiles. This means that one can only interpret the difference in the pupil size in response to the information on the different scenario types within each quartile. Recent behavioral research has shown that depressive symptoms are related to a reduced integration of positive information rather than to a hypersensitivity to novel negative information (Everaert et al., 2018; Kube et al., 2019b; Kube and Glombiewski, 2022; Kube, 2023). Therefore, we interpret the enlarged difference in the pupil response to the last information on the two scenario types in the last BDI-II quartile in this direction as well. Nevertheless, to be able to entirely understand the mechanisms underlying the difficulties of depressed individuals to integrate positive information, future research is needed. Specifically, future research should aim to disentangle the effects of valence and (un-) expectedness of new information by using a fully balanced design of the emotional BADE task, comprising disconfirming-the-positive and confirming-the-positive in addition to the DTN and CTN scenarios used in the present study.

As our initial aim in this proof-of-concept study was to explore the applicability of the emotional BADE task in the context of eye-tracking, the decision for a relatively small sample size was intentional. However, we acknowledge the limitations posed by the small sample size, in particular the challenges of detecting significant effects, even if they were potentially substantial. Moreover, the large proportion of highly educated university students and the underrepresentation of individuals with diagnosed depression in our sample raises concerns about the generalizability of our findings. Follow-up studies should therefore look at more diverse and larger samples including clinically depressed patients. Group comparisons between healthy individuals and those with more severe depression will be crucial in order to further specify the current results regarding the blunted processing of unexpected positive information. Nevertheless, the results obtained in our sample offer valuable initial insights, suggesting that the experimental paradigm is applicable to our research question and can effectively be utilized to explore differences in pupillary responses between depressed and healthy individuals. As part of an enhanced sample design, it is imperative to systematically account for potential confounding variables in the assessment of the pupillary response to emotional stimuli. Therefore, in a forthcoming study, we intend to carefully monitor potential confounders such as the current positive or negative affect or the use of antidepressant medication and introduce stricter exclusion criteria such as drug abuse. Additionally, it is planned to match the healthy and depressive subsamples in terms of age and gender in order to increase internal validity.

A further limitation is that this study was not pre-registered as its main goal was to test the methodology (i.e., the adapted BADE task and the pupillary response as a psychophysiological measure of predictive errors provided by it) used to investigate psychophysiological mechanisms of biased belief updating in relation to depressive symptoms. Since this proof-of-concept largely confirmed the suitability of this methodology, future research building on it is required to pre-register and rigorously test the hypothesis of a reduced pupillary response in response to novel positive information in clinically depressed individuals.

A final limitation is that the number of interpretations presented for each interpretation type in one scenario (i.e., one absurd, one true, but two lure interpretations) could have influenced the participants' plausibility ratings. Since the two lure interpretations lead to a predominance of interpretations with a certain valence (e.g., positive in the CTN condition), this may have biased the plausibility ratings in the respective direction, at least in the beginning of a scenario. This could also be an explanation of the plausibility ratings in the CTN condition, indicating the highest plausibility for the positive interpretations even though the initial information on these scenarios was negatively valanced (see Section 3.2). Another effect of presenting two lure interpretations with the same valence could be that attentive participants could infer the outcome of a scenario. Eventually, participants could have learned over the course of the task, that scenarios presented with two negative interpretations always had a positive ending and vice versa. This predictability could have led to a reduced surprise effect of the final information on the scenarios presented toward the end of the task. Even though the follow-up interview did not indicate that participants were aware of this relationship, we plan to delete one lure interpretation in a future version of the task. This way we can ensure that the ending of the scenarios is not predictable by the valence of the interpretations presented.

### 4.2 Conclusion

Despite these limitations, the present proof-of-principle study provides evidence that the adapted form of the emotional BADE task can be suitable for examining psychophysiological parameters such as pupil dilation in addition to behavioral measures in a context which we deem more relevant to depression, i.e., a social context. At the behavioral level, we could provide further evidence for the difficulties of people with elevated depressive symptoms to adjust established negative interpretations after disconfirmatory positive information. Based on the common interpretation of pupil dilation as a marker of processing prediction errors, analyses of pupillary changes suggested an impaired processing of unexpected positive information indicated by decreased pupil dilations associated with higher depression scores. Consistent with the theoretical assumptions of recent predictive processing models, this may account for the failure of depressed individuals to integrate novel positive information.

## Data availability statement

The raw data supporting the conclusions of this article will be made available by the authors, without undue reservation.

## Ethics statement

The studies involving humans were approved by Local Ethics Committee of the University of Koblenz-Landau (reference number: LEK-310). The studies were conducted in accordance with the local legislation and institutional requirements. The participants provided their written informed consent to participate in this study.

## Author contributions

AS, SK, and TK developed the study concept. AS, SK, JE, and TK contributed to the study design. Testing and data collection were performed by AS and SK. AS and SK performed the data analysis and interpretation, in consultation with the other authors. AS drafted the manuscript. All other authors provided critical revisions and approved the final version of the manuscript for submission.
